# A dose- and time-dependent effect of oxythiamine on cell growth inhibition in non-small cell lung cancer

**DOI:** 10.1007/s11571-021-09725-7

**Published:** 2021-11-09

**Authors:** Lin Bai, Hui-li Zhu

**Affiliations:** Department of Respiratory Medicine, Huadong Hospital, Fudan University, 221 West Yan’an Road, Shanghai, 200040 China

**Keywords:** NSCLC, Thiamine-dependent enzymes, Oxythiamine, Apoptosis, Cell cycle

## Abstract

The high mortality rate of non-small-cell lung cancer (NSCLC) is mostly due to the high risk of recurrence. A comprehensive understanding of proliferation mechanisms of NSCLC would remarkably contribute to blocking up the invasion and metastasis of tumor cells. In our previous study, the remarkable decreased activity of Thiamine-dependent enzymes (TDEs), involving in intermediary metabolism responsible for energy production of tumor, was found under conditions of thiamine deficiency in vivo. To explore the effect of Oxythiamine (OT), a TDEs antimetabolite, on cell growth, we co-cultured A549 cells with OT in vitro at various doses (0.1, 1, 10 and 100 μM) and time periods (6, 12, 24 and 48 h) and subsequent cell proliferation and apoptosis assays were performed respectively. Our findings demonstrated that A549 cells proliferation was significantly downregulated by OT treatment in a progressively dose as well as time dependent manner. Inhibition of TDEs resulted in antagonism of lung cancer growth by inducing cells to cease the cycle as well as apoptotic cell death. We concluded a critical role of OT, a TDEs antagonistic compound, indicating the potential target of its practical use.

## Introduction

Non-small-cell lung cancer (NSCLC), classified by histologic characters as adeno-, and squamous-carcinoma, and large cell carcinoma, accounts for the biggest proportion of lung tumor (Ji et al. 2011). The 5 years survival rate in NSCLC patients is less than 15% (Reck and Rabe 2017; Howlader et al. 2020) More evidence suggests that the low survival rate of NSCLC partly results from the low diagnostic rate as well as high risk of recurrence. It will be valuable to better understand the underlying proliferation mechanisms of lung cancer, which would contribute to blocking up the invasion and metastasis of cancer cells.

Metabolism is essential for tumor cells proliferation and division. A comprehensive ingredient of nutrients, known as glucose and micronutrients essential for enzyme cofactors is needed to meet the high metabolic demands of cancer cells. Thiamine pyrophosphate (TPP) is the most essential one involving in several metabolic ways, such as Pentose phosphate pathway (PPP) non-oxidative pathway, glucose metabolism tricarboxylic acid cycle (TCA), and amino acid metabolism. Previous reports showed that thiamine-dependent anabolic pathways are involved in the adjustment of cancer cell metabolism via thiamine (vitamin B1)-dependent enzymes (TDEs) (Zastre et al. 2013; Chauvier et al. 2017; Boros et al. 2000; Ortigoza-Escobar et al. 2017; Peterson et al. 2020).

Oxythiamine (OT), an antagonism of thiamine, disturbs with the enzymes of thiamine pyrophosphate dependent transketolase functioning and can reduce nucleic acid biosynthesis through decreases in ribose 5-phosphate (R5P) and NADPH synthesis (Platell et al. 2000; Cairns et al. 2011; Liu et al. 2020; Ramos-Montoya et al. 2006) TDEs is of great increased expression in Oral Squamous Cell Carcinoma and its antagonistic compounds would be of meaningful value to sensitize tumor cells to conventional oral squamous cell carcinoma therapies (Grimm et al. 2016). Carmela et al. found that nuclear transketolase (TKT) positivity is increased in ovarian tumors with abdominal metastases, and be concerned with low survival. Inhibited TKT by OT administration resulted in suppressing the growth of ovarian cancer cells (Ricciardelli et al. 2015). Moreover, oxythiamine can enhance the efficacy of imatinib in leukemia cells (Zhao et al. 2010).

However, no existing studies have clarified OT expression and its potential role in NSCLC. Recently, our group has explored the neoplastic metabolite changes treated with OT in mice, using the stage of 1D1H nuclear magnetic resonance (Lu et al. 2015). The metabolites in tumor associated with TDEs underwent considerable change between OT and control groups, indicating concentration dependence and enzyme specificity. Our current study aimed to subsequently investigate the effect of OT on cell growth in lung adenocarcinomic cell lines.

## Materials and methods

### Cell cultures and reagents

Lung carcinoma A549 was a kind gift from the ZhongShan Hospital, Fudan University, Shanghai and seeded at a density of 3 to 5 million cells. Cells were grown in DMEM, plus 10% fetal bovine serum in a designed humidified environment with 5% CO_2_ at 37 °C. The culturing medium was changed every 48 h, and cells were passaged upon confluence reaching to 80%. All cell-based assays were performed during exponential cellular growth phase.

Oxythiamine-Hcl and Thiamine and were obtained from Sigma (USA). Phosphate-buffered saline, DMEM and trypsin–EDTA were purchased from Ginuo (China). FBS was obtained from Gibco (Gibco, USA). Tissue culture dishes were obtained from Corning. All the culturing processes were performed in a CO_2_ Incubator (Thermo Scientific, USA).

### Cell proliferation assays

The assays were performed using the reagents WST-8 (Dojindo, Japan). Briefly, A549 Cells were seeded at 20,000 cells/well for 24 h and then treated with different levels of oxythiamine (0–100 μM) for 6, 12, 24 and 48 h. Viability was documented, 10 μl of WST-8 reagents were added into wells and incubated for 60 min. The absorbance was collected at 450 nm by spectrophotometer (TECAN, Austria). Each group was repeated for 6 times, and the experiment was repeated for three times.

### Cell cycle analysis

A549 cells were treated with different dosages of Thiamine (10 μM) or Oxythiamine (0–100 μM) for 24 and 48 h. All the cells were trypsinized, washed and then fixed in 70% ethanol at a density of 1 million cells/ml before preserving in − 20 °C for no less than 24 h. After overnight incubation at 4 °C in ethanol, cells were washed and suspended in 0.5 mL DNA staining solution [50 μg/ml PI and 100 μg RNaseA in PBS (BD, USA)] 15 min at room temperature before flow cytometry. Finally, samples were analyzed by Cell-Quest software (Becton–Dickinson). The experiments were carried out three times on separated days.

### Apoptosis assay

Cell apoptosis was measured following the instruction of Annexin V-FITC/PI Kit (BD Biosciences). Briefly, A549 cells were seeded at the density of 100,000 cells/well and the medium was refreshed with the supplementary of 0.1–100 μM oxythiamine 24 h later. After 24 and 48 h, the cells were trypsinized, washed and then collected by centrifugation. Subsequently, the cells were co-cultured with 5 ml Annexin V-FITC and 10 ml PI in a binding buffer for 30 min at room temperature, and resuspended in the same buffer. The detection of cells upon all the apoptotic conditions were performed by FACS (Biosciences). This assay was performed in triplicate.

### Statistical analysis

All values were listed as mean ± SD. Data were analyzed by ANOVA followed by Tukey’s multiple comparison tests using Sigmaplot 11.0 software. *p* < 0.05 was represented as significant difference.

## Results

### Inhibition of cell proliferation by oxythiamine

A549 is a classic model system of pulmonary tumor cells. To determine the effects of oxythiamine on lung cancer cells, we firstly observed the influence of oxythiamine on the growth of A549.

**Fig. 1:**
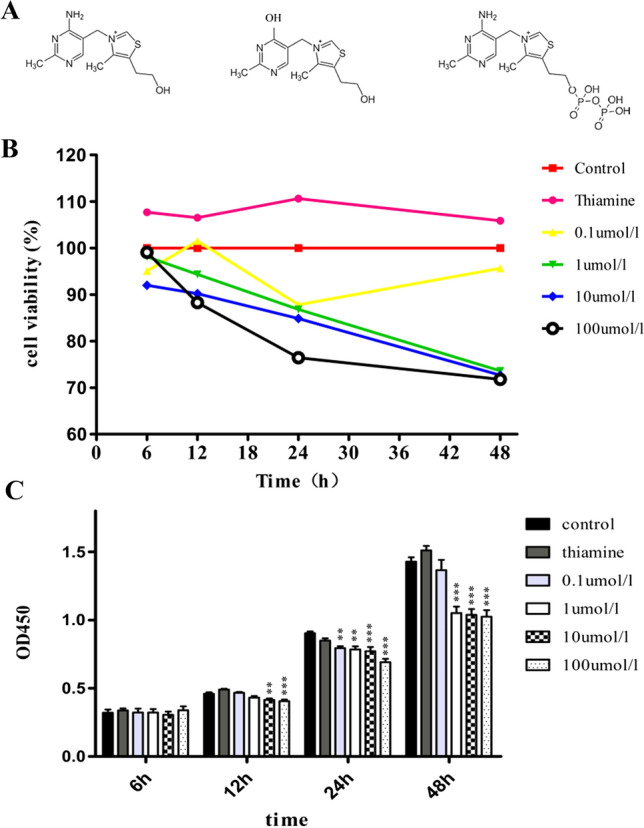
**A** Chemical structures of thiamine (left), oxythiamine (middle) and the active co-enzyme thiamine pyrophosphate (right). **B** and **C** Effects of OT on the proliferation of A549 cells. Dose–response and Time-course: A549 cells were cultured in medium supplemented or not (control) with OT from 0.1 to 100 μM for 6, 12, 24 and 48 h. Then, cell viability was measured by CCK-8 assay. All data from three separate experiments are presented as mean ± SD. *p < 0.05; **p < 0.01;***p < 0.001

We performed dose and time related experiments. A549 cells were co-cultured with step-increasing levels of oxythiamine (from 0.1 to 100 μM) for 6–12 h and cell proliferation was subsequently assessed by CCK-8 assay. As reported in Fig. 1B, C, oxythiamine contributes to a dosage relevant decrease of viability of A549 cells (*p* < 0.05) with the initial dose of 10 μM for 12 h. In time-course experimental sets, A549 cells were exposed to oxythiamine 100 μM for 6, 12, 24 and 48 h, after which cell proliferation was assessed by CCK-8 kit. Oxythiamine contributes to a significant reduction of A549 cell viability of 11.7%, 23.6%, and 28.2% at 12, 24, and 48 h, respectively. Taken together, all the findings suggested an anti-proliferative effect of oxythiamine on A549 cells (Fig. 1B, C).

### Effect of oxythiamine on cell division cycle

For the next step, to investigate the inhibition effect of Oxythiamine on A549 cells, distribution of cells upon all the growth cycle was determined. Inducing cancer cells to cease the cycle, known as cell-cycle arrest, is supposed to be one of the most useful interventions to restrain the spread of tumors.

**Fig. 2:**
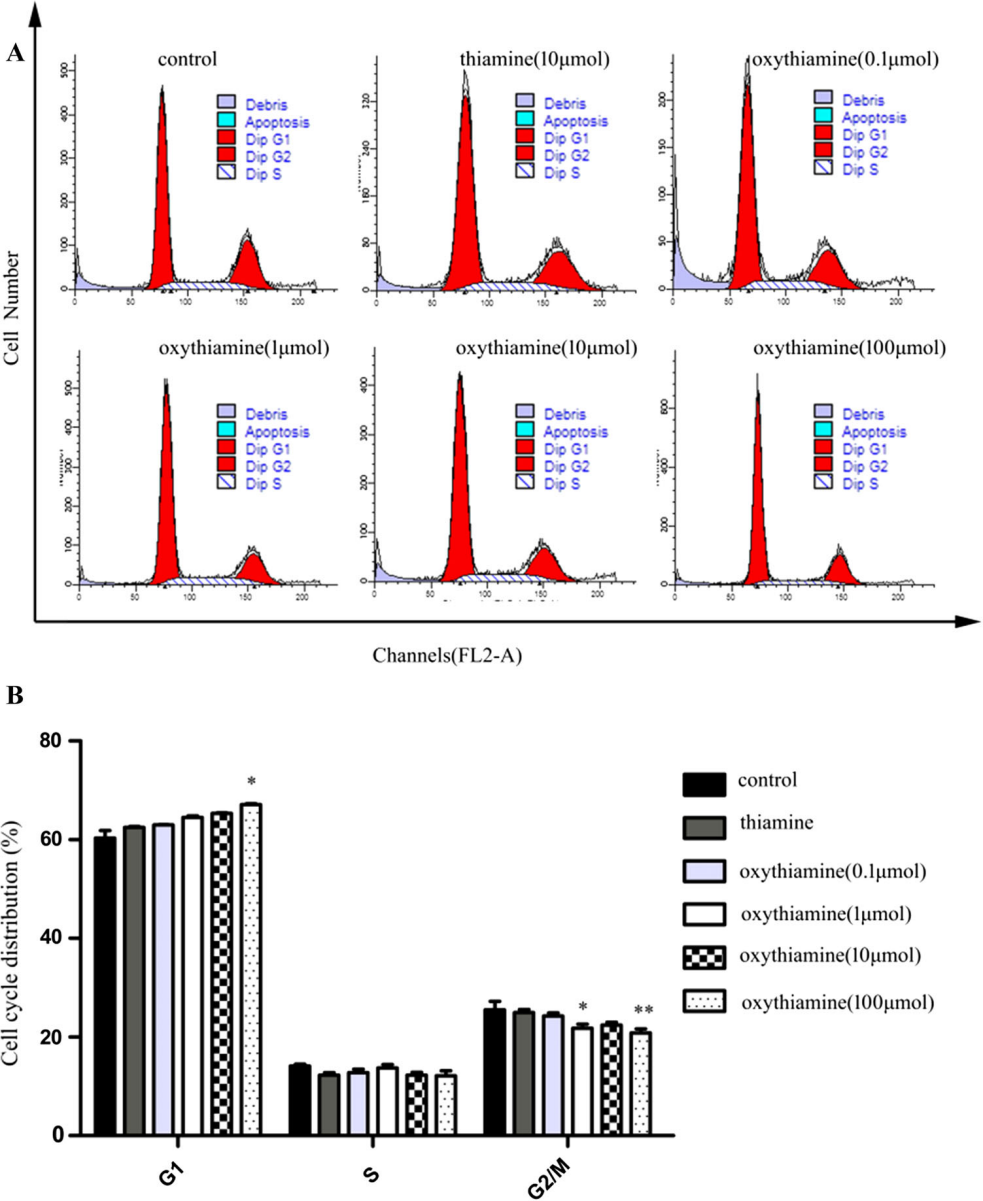
Effects of OT on cell cycle in A549 cells. Cells were cultured in medium supplemented or not (control) with OT 0.1–100 μM for 24 h. Then, FACS analysis was performed. OT induced cell cycle arrest at G1 phase. **A** Representative FACS histograms of propidium iodide stained A549 cells are shown. **B** The percentage of each cell-cycle phase is indicated. *p < 0.05; **p < 0.01; ***p < 0.001

**Fig. 3:**
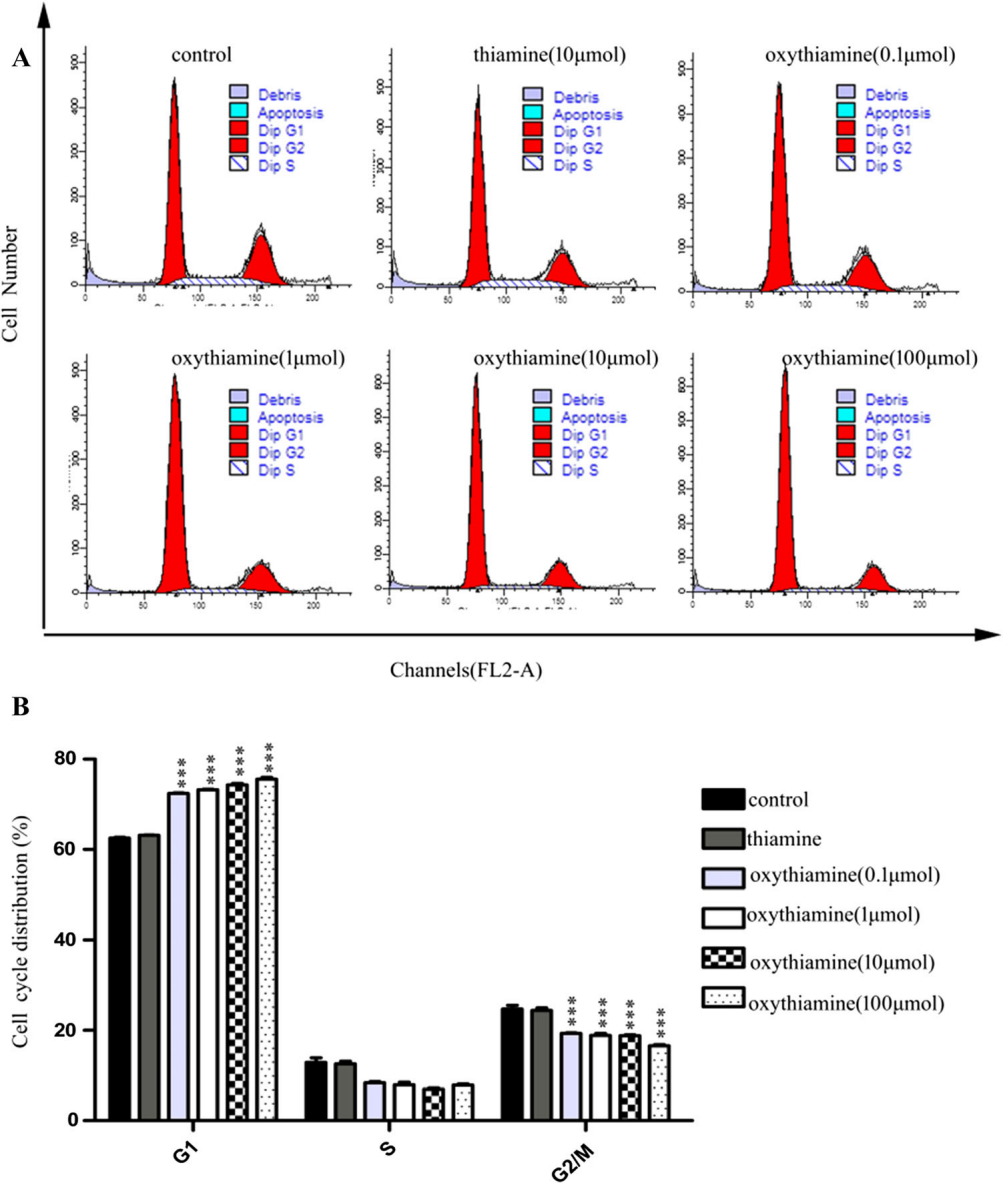
Effects of OT on cell cycle in A549 cells. Cells were cultured in medium supplemented or not (control) with OT 0.1–100 μM for 48 h. Then, FACS analysis was performed. OT induced cell cycle arrest at G1 phase. **A** Representative FACS histograms of propidium iodide stained A549 cells are shown. **B** The percentage of each cell-cycle phase is indicated. *p < 0.05; **p < 0.01; ***p < 0.001

As shown in Figs. 2 and 3, at designed time points (from 24 to 48 h), among Oxythiamine-treated (0.1–100 μM) A549, the proportion of G1 phase is remarkably higher than that of control and Thiamine treated cells, while the proportion of G2/M phase is decreased. Our results indicate that oxythiamine (100 μM, 48 h) influences A549 cell cycle with an increase of 13.15% in G1 phase and a decrease of 8.13% in G2/M phase, indicating that oxythiamine arrests the cell growth in G1 phase of cell cycle.

### Cell apoptosis related to oxythiamine

**Fig. 4:**
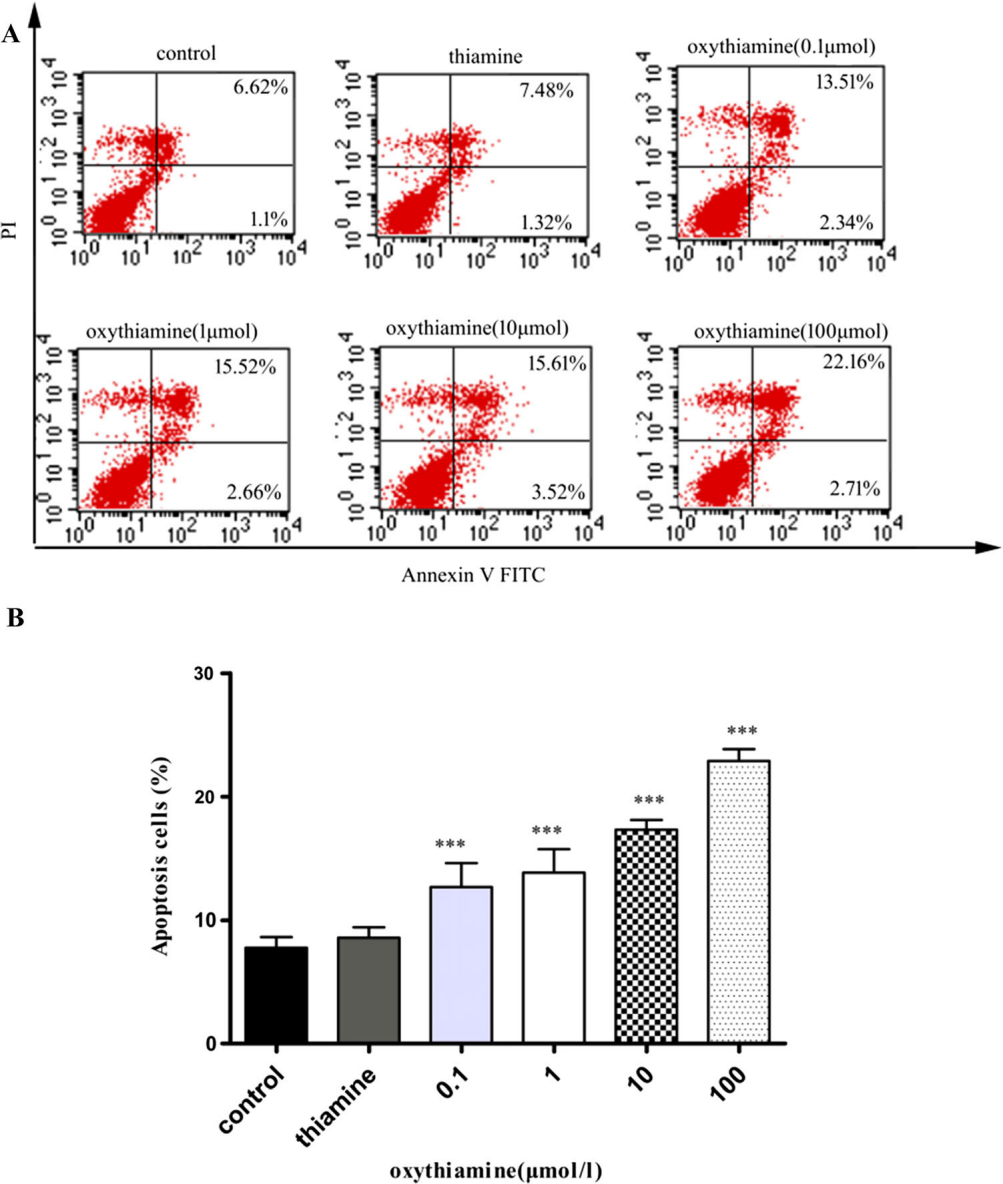
Effects of OT on cell apoptosis in A549 cells. Cells were cultured in medium supplemented or not (control) with OT 0.1–100 μM for 24 h. Then, FACS analysis was performed. **A** Representative dot plots V-FITC (x-axis)/PI (y-axis) of both annexin V-FITC and propidium iodide stained A549 cells. The percentage of early apoptotic cells (LR) and late apoptotic cells (UR) is indicated. **B** The percentage of apoptotic A549 cells which treated with OT (0.1–100 μM) were 12.667 ± 4.735%, 13.838 ± 4.661%, 17.325 ± 1.964% and 22.903 ± 2.356%, respectively. *p < 0.05; **p < 0.01; ***p < 0.001

**Fig. 5:**
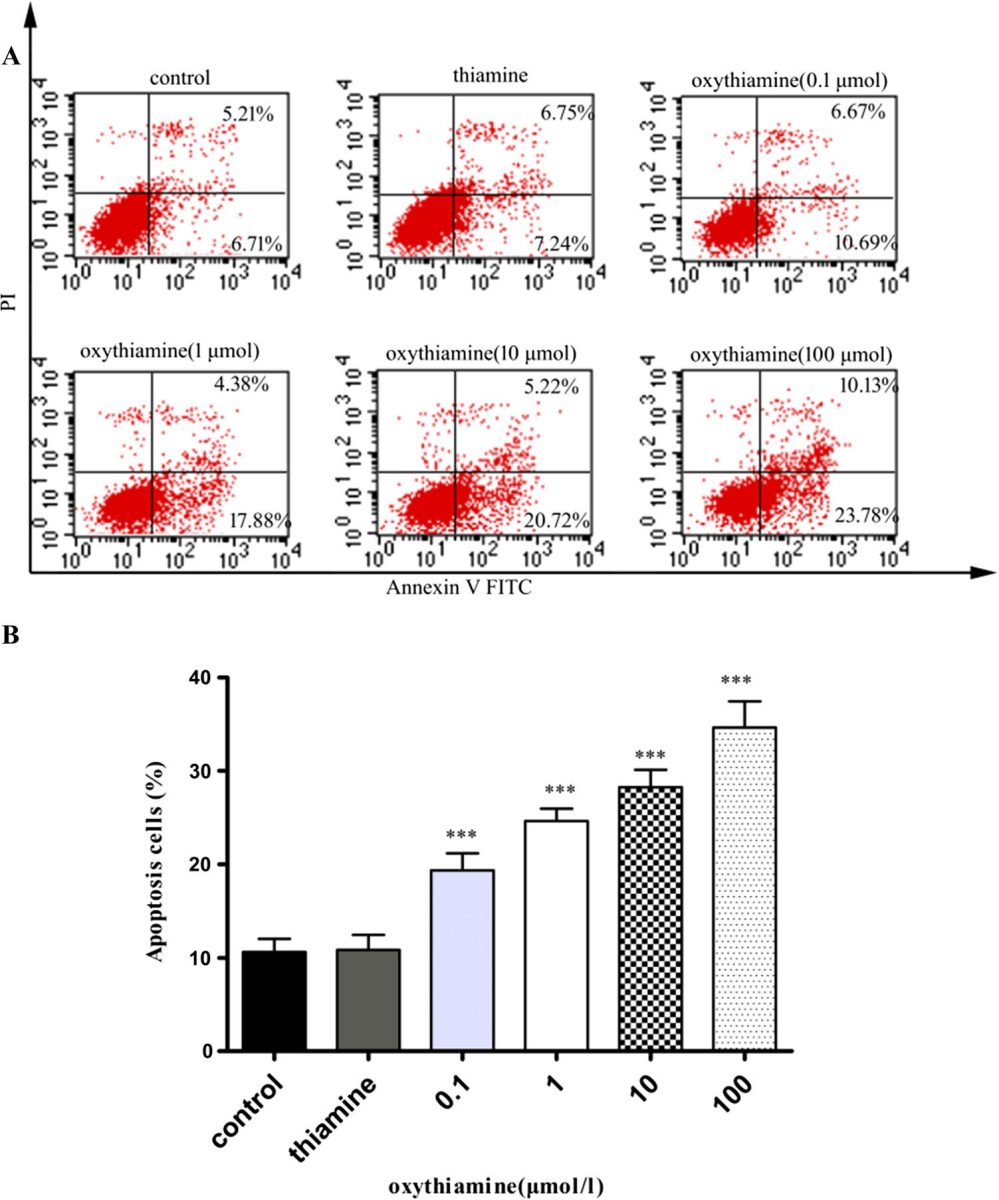
Effects of OT on cell apoptosis in A549 cells. Cells were cultured in medium supplemented or not (control) with OT 0.1–100 μM for 48 h. Then, FACS analysis was performed. **A** Representative dot plots V-FITC (x-axis)/PI (y-axis) of both annexin V-FITC and propidium iodide stained A549 cells. The percentage of early apoptotic cells (LR) and late apoptotic cells (UR) is indicated. **B** The percentage of apoptotic A549 cells which treated with OT (0.1–100 μM) were 19.352 ± 4.522%, 24.655 ± 3.236%, 28.290 ± 4.494% and 34.638 ± 6.877%, respectively. *p < 0.05; **p < 0.01; ***p < 0.001

Existing evidence supports molecular levels fluctuate during the apoptosis process, including membrane phosphatidylserine externalization (Zargarian et al. 2017; Birge et al. 2016). This special change would be demonstrated with the help of annexin-V labeled with FITC (Subba Rao et al. 2017). To study the mechanisms of oxythiamine in the growth inhibition of lung cancer cells, apoptosis analyses were performed on A549 cells 24 and 48 h after drug exposure, using the flow cytometric analysis of Annexin V-FITC/PI. In order to demonstrate if apoptosis happens after oxythiamine treatment, we labeled A549 cells with Annexin V and PI and then observed apoptosis by FACS. As shown in Figs. 4 and 5, apoptotic A549 cells were obvious after 24 h of 0.1 μM oxythiamine administration (15.44% compared to control group) and further increased at 48 h (31.45% compared to control group). Taken together, all the findings indicate that oxythiamine inhibits the growth of A549 cells by initially slowing-down cell cycle and by leading to apoptosis.

## Discussion

In our study, we first identified TDEs inhibitors OT significantly reduced proliferation of A549 cells, especially in a dose- and time-relevant manner. Our findings indicated that OT induce cell cycle arrest in G1 phase and apoptosis also with dose- and time-dependent, which is in coincidence with our previous results showing the metabolites in tumor associated with TDEs exhibiting dosage dependence and enzyme specificity. All these findings supported the hypothesis that thiamine-dependent enzymes would be an important regulator for NSCLC, and the antagonist OT has the potential to be a new target of tumor therapy.

Since Warburg firstly identified, non-oxidative glucose metabolism through the PPP has been proved to promote cancer cell growth and be controlled by thiamine-dependent transketolase enzyme reactions (Halma et al. 2017). Different treatments using either thiamine antagonists (OT) or thiamine deprivation have been suggested to eliminate thiamine from tumor cells. Also, oxythiamin in conjunction with other interventions would be a valuable therapeutic strategy for drug-resistant cancers (Ramos-Montoya et al. 2006; Zhao et al. 2010). However, the responses of different tumors to oxythiamin change widely in vitro. However, the responses of different cancer cells to oxythiamin differ greatly, both in vitro and in vivo. For instance, oxythiamin decreased the viability of human colon adenocarcinoma cells at 5400 μmol (IC50) while that in MIA pancreatic carcinoma cells was achieved at concentrations three orders of magnitude lower, 0.25 μmol. In our study, oxythiamine treatment at a level of 10 μmol (cultured 12 h) resulted in a significant decrease in NSCLC proliferation, which is in accordance with studies on other cancers (Liu et al. 2010; Lin et al. 2011; Zhang et al. 2010). However, other studies on Lewis Lung Carcinoma cells failed to find a benefit on proliferation but a remarkable inhibition of invasive capability with a concentration of oxythiamine (20 μmol) in vitro. Daily administration of 500 mg/kg OT considerably inhibited metastases (Yang et al. 2010) and N3’-pyridyl thiamin, as a thiamin antagonist, totally inhibited the expression of transketolase in HTC-116 cell lines without any effect on the cancer cell growth (Thomas et al. 2008). These controversial interpretations may come from different experiment design, tumor cell line, and culture medium with or without super-physiological levels of thiamine. It emphasized the requirement for following works on the participation of several thiamine-relevant enzymes among several neoplastic cells.

On the other hand, Rais et al. demonstrated that inhibiting TKT reactions by oxythiamine administration resulted in the arrest of Ehrlich’s ascitic tumor cells in the G1 phase of cell cycle (Halma et al. 2017). This could be explained by the fact that TKT levels increase during G1/S phase. Notably, majority of studies on anticancer therapeutic strategies with thiamin antagonists came from the concept of what neoplastic metabolic reprogramming were needed (Ramos-Montoya et al. 2006). More interestingly, restoration of PDH activity in cancer cells has been shown to promote apoptosis and is suppressed in cancer due to downregulation and overexpression of PDK isoforms (Lu et al. 2011; Hur et al. 2013; Baumunk et al. 2013; Sun et al. 2019). We found in A549 cell line, OT can block G1 phase, retard cell proliferation, and induce cell apoptosis, with time and concentration dependent manner. Accordance with our previous reports that PDH was restrained by 150 mg/kg OT in mice (Lu et al. 2015), we therefore assume that OT may promote apoptosis via adjust the expression of PDH/PDK or TKT in tumor cells. Oxythiamine also inhibited phosphorylation of several proteins during the cell cycle such as Hsp27 (Thomas et al. 2008). Other evidence demonstrated that Oxythiamine has more recently been shown to alter the dynamics of cellular protein expression in MIA PaCa-2 pancreatic cells by interrupting the rates of de novo protein synthesis involved in several apoptotic signaling pathways (Wang et al. 2013). All the findings suggest that oxythiamine have the possibility to influence other signaling pathways. Little is known so far about the significance of TDEs, which requires more future investigations.

However, our study have limitations. The results do not indicate the role of OT in other cell lines of NSCLC. More researches are required to better explain the molecular mechanisms of TDEs in cancer pathogenesis.

## Conclusion

In conclusion, our study demonstrates that TDEs plays an essential role in the proliferation of lung adenocarcinomic cells. Metabolic targeting of thiamine-relevant enzymes by oxythiamine, with a dose- and time-dependent manner, indicates the potential of its practical use in NSCLC therapy.
